# Repeat Fall Risk in Geriatric Patients After Fall-Induced Head Trauma

**DOI:** 10.7759/cureus.45056

**Published:** 2023-09-11

**Authors:** Scott M Alter, Brandon W Knopp, Joshua J Solano, Patrick G Hughes, Lisa M Clayton, Richard D Shih

**Affiliations:** 1 Department of Emergency Medicine, Florida Atlantic University Charles E. Schmidt College of Medicine, Boca Raton, USA; 2 Department of Emergency Medicine, Delray Medical Center, Delray Beach, USA

**Keywords:** emergency medicine, readmissions, head trauma, falls, geriatrics

## Abstract

Introduction

There are many known risk factors for falls, with poor health and physiologic decreases in function as the major contributors to fall risk in older adults. However, risk factors for repeat falls after initial ED discharge are not well-described. This study seeks to prospectively investigate risk factors for short-term repeat falls in geriatric ED patients with fall-related head trauma who do not require hospital admission.

Methods

This is a prospective study of patients aged 65 years and older with fall-related head trauma who presented to the EDs of two community level I trauma centers. Patients were excluded for intracerebral hemorrhage, admission during initial ED visit, or death in the hospital. Patients were followed for 14 days. Patient characteristics, repeat ED visits, and reason for returns were noted.

Results

About 2,143 patients were identified as meeting the inclusion criteria. Within 14 days of the initial presentation, 14.1% of patients returned to the ED, with 8.3% presenting with a complaint related to the initial trauma and 2.6% with a new injury. Patients with comorbidities of dementia (OR 3.02, 95% CI, 1.72-5.33, p<0.001), stroke (OR 2.12, 95% CI, 1.05-4.27, p=0.031), and smoking (OR 4.27, 95% CI,1.76-10.37, p<0.001) were significantly more likely to sustain a new injury leading to a repeat ED visit within 14 days.

Conclusions

After an ED visit due to a fall, over one in 10 patients will re-present to the ED due to a new injury or sequelae from the initial fall. In the immediate period after a fall, enhanced outpatient follow-up or risk mitigation strategies should be considered to lessen return visits and decrease morbidity.

## Introduction

Falls are a significant cause of morbidity and mortality in the geriatric population. In the United States, approximately one in four adults older than 65 years of age report falling each year, resulting in an estimated three million ED visits annually [1,2]. Falls are the leading cause of accidental deaths in this population, accounting for 30,000 deaths annually [1,3]. Specific risk factors for falling include visual impairment, polypharmacy, balance and gait instability, depression, dizziness, vertigo, orthostasis, cognitive impairment, diabetes, reduced muscle strength, and other physical and mental impairments [4,5]. However, risk factors for recurrent falls are less understood.

In geriatric patients who present to the ED after fall-related head trauma, there is a significant risk of injury due to repeat falls with an increasing risk of associated morbidity and mortality proportional to each subsequent fall [6,7]. In particular, patients with a traumatic brain injury have a 30-day readmission rate between 8.9% and 14.4% [8,9]. To reduce this high rate, additional interventions to prevent ED return visits, such as increased outpatient follow-up and the Centers for Disease Control and Prevention's Stopping Elderly Accidents, Deaths, and Injuries (STEADI) program, have been developed [10]. While all patients are recommended to partake in these activities, access may be restricted due to limited resources.

Patients not hospitalized following their ED presentation for head trauma may have had no injury or minor injuries not requiring admission. However, these patients may have an increased risk of repeat injuries. Identifying characteristics to predict future falls, with the subsequent targeted implementation of fall-prevention strategies, could help reduce morbidity and mortality.

This study seeks to investigate the characteristics of geriatric ED patients who do not require hospital admission after an initial fall-related head injury but return to the ED within 14 days. Additionally, risk factors for recurrent falls will be determined.

This research was presented at the Florida College of Emergency Physicians Symposium by the Sea in Bonita Springs, Florida, on August 5-6, 2022.

## Materials and methods

Study design and setting

This is a prospective cohort study of patients who presented to the EDs of two university-affiliated community hospitals and level I trauma centers with annual ED volumes of 50,000 and 69,000. These facilities are the only trauma centers serving one South Florida county. The study received approval from the institutional review board of Florida Atlantic University (1326154).

Selection of participants

The study enrollment spanned from August 2019 to August 2020 and included participants aged 65 years and older who presented to the ED with head trauma due to a fall. Patients were excluded if they were injured more than 24 hours before presentation, had an intracranial hemorrhage, were transferred from another hospital, were discharged into hospice care, were admitted during the initial ED visit, or sustained fatal injuries.

All patients with a CT head performed in the ED for suspected head injury or diagnosed with a head injury by ICD-10 code (between S00 and S09) were identified daily during the study enrollment period. Trained research assistants screened and enrolled patients if they met the study criteria.

Data collection

Research assistants blinded to the study hypothesis performed chart reviews of ED physicians and nurse notes upon patients’ initial presentations. Independent variables collected included demographic characteristics (age, gender, and ethnicity), past history (medical history, social history, anticoagulant, and antiplatelet use), presenting symptoms, physical examination findings, blood alcohol level, trauma team activation status, and hospital arrival method (ambulance or private vehicle). Symptoms, physical examination findings, and blood alcohol levels were only included if the items were documented in the medical chart.

After 14 days from each patient’s initial visit, the electronic medical record was queried for any repeat ED visits. A chart review was performed for each subsequent visit. Research assistants categorized the reason for each repeat visit as a new traumatic injury, a new medical complaint, or sequelae of the initial fall. New trauma was further subdivided by mechanism as either a second fall or a non-fall injury. Return visits due to sequelae of the initial fall without new trauma were subcategorized as expected follow-up (e.g., suture removal), persisting head trauma symptoms since the initial ED visit, new head trauma symptoms not present during the initial ED visit, sent by an outpatient clinician without patient complaint, and other complaint related to initial fall but not related to head trauma (e.g., arm pain). The Florida Bureau of Vital Statistics death registry was also queried for each patient to determine if they had died since the initial fall.

All data was compiled and inputted into the REDCap database system with real-time parameter validation. Principal investigators and research assistants communicated regularly to discuss and resolve study-related issues, minimizing inclusion discrepancies.

The primary study outcome was a repeat ED visit for a new traumatic injury within two weeks of the initial head injury. The secondary outcome measure was the primary chief complaint for the repeat ED visit.

Statistical analysis

Frequencies were computed for the number of return ED visits and for the reasons for return visits. The rates of ED returns for patients with new traumatic injuries within 14 days of the initial falls were calculated for patients with and without each independent variable. Comparisons were performed using the chi-squared test. Odds ratios were also calculated by univariate analyses. Significance was defined as a p-value of <0.05. Analyses were performed using SPSS Statistics version 27.0 (IBM Corp. Released 2020. IBM SPSS Statistics for Windows, Version 27.0. Armonk, NY: IBM Corp).

## Results

Characteristics of study subjects

During the enrollment period, 2,143 patients were identified as meeting the study inclusion criteria. The average age was 82.5 ± 8.5 years and 60.9% were female. The patients were 91.1% White, 4.3% African American, 3.5% Hispanic, and 1.1% other races.

Main results

Within 14 days of the initial ED presentation, 14.1% (302) of patients returned to the ED. Of these, 277 (12.9%) had one visit, 21 (1.0%) had two visits, three (0.1%) had three visits, and one (<0.1%) had four visits. During the first repeat visit to the ED, 177 patients (58.6%) had a complaint related to the initial fall without a new injury, 69 (22.8%) had a new medical complaint, and 56 (18.5%) had experienced a new injury (Table 1). The mechanism of the new injury was a second fall for 54 (96.4%) and a blunt injury not related to a fall for the remaining 2 (3.6%). Overall, 24 patients (1.1%) died within 14 days of the initial ED visit.

**Table 1:** Reasons for first repeat ED visits

Reason for the first return visit	N (%)	
Sequelae of initial fall	177 (58.6%)	
Expected follow-up		126 (71.2%)
Persisting head trauma symptoms		13 (7.3%)
New head trauma symptoms		12 (6.8%)
No symptoms but sent back after routine follow-up		3 (1.7%)
Other complaints not related to head trauma		23 (13.0%)
New medical complaint	69 (22.8%)	
New traumatic injury	56 (18.5%)	
Second fall		54 (96.4%)
Non-fall injury		2 (3.6%)

No significant differences in 14-day ED return rates were found based on demographic characteristics including gender, age, or race. Patient characteristics including past medical history, social history, anticoagulant use, and antiplatelet use are shown in Table 2. Patient comorbidities including dementia (OR 3.02, 95% CI, 1.72-5.33, p<0.001) and stroke (OR 2.12, 95% CI, 1.05-4.27, p=0.031) were associated with a significantly increased risk of ED return within 14 days. Among social factors, tobacco use was the only variable associated with an increased patient risk of ED return within 14 days (OR 4.27, 95% CI,1.76-10.37, p<0.001). Neither anticoagulant nor antiplatelet medications altered the risk of a 14-day ED return. Patient characteristics relating to initial ED presentation are shown in Table 3. No physical exam findings evaluated in this study were found to alter the risk of a 14-day ED return.

**Table 2:** Fourteen-day ED return rates for patients with new traumatic injuries by past medical history, social history, and anticoagulant/antiplatelet use

Factor	Factor present	Factor absent	Odds ratio (95% CI)	p-value
Female	31 (2.4%)	25 (3.0%)	1.27 (0.74-2.16)	0.385
Age >80 years	39 (2.8%)	17 (2.2%)	1.31 (0.73-2.32)	0.363
Atrial fibrillation	12 (3.5%)	44 (2.4%)	1.43 (0.75-2.74)	0.276
Cancer	8 (3.3%)	48 (2.5%)	1.33 (0.62-2.85)	0.458
Chronic obstructive pulmonary disease	4 (4.0%)	52 (2.5%)	1.60 (0.57-4.50)	0.373
Congestive heart failure	2 (2.8%)	54 (2.6%)	1.07 (0.26-4.47)	0.929
Coronary artery disease	8 (3.9%)	48 (2.5%)	1.59 (0.74-3.41)	0.229
Deep venous thrombosis	0 (0%)	56 (2.7%)	-	0.308
Dementia	19 (5.9%)	37 (2.0%)	3.02 (1.72-5.33)	<0.001
Diabetes	10 (2.9%)	46 (2.6%)	1.16 (0.58-2.32)	0.679
Hypertension	35 (3.2%)	21 (2.0%)	1.65 (0.95-2.85)	0.071
Peripheral vascular disease	1 (5.6%)	55 (2.6%)	2.21 (0.29-16.93)	0.432
Pulmonary embolism	0 (0%)	56 (2.6%)	-	0.401
Stroke	10 (4.9%)	46 (2.4%)	2.12 (1.05-4.27)	0.031
Valve replacement	0 (0%)	56 (2.6%)	-	0.524
Alcohol use	9 (3.2%)	47 (2.5%)	1.29 (0.62-2.66)	0.492
Tobacco use	6 (9.5%)	50 (2.4%)	4.27 (1.76-10.37)	<0.001
Anticoagulant use	16 (3.1%)	40 (2.5%)	1.25 (0.69-2.25)	0.457
Antiplatelet use	23 (3.1%)	33 (2.3%)	1.36 (0.79-2.33)	0.269

**Table 3:** Fourteen-day ED return rates for patients with new traumatic injuries by initial ED presentation and findings

Factor	Factor present	Factor absent	Odds ratio (95% CI)	p-value
Glasgow coma scale <15	8 (4.7%)	48 (2.4%)	1.98 (0.92-4.26)	0.075
Altered mental status	0 (0%)	18 (2.7%)	-	0.215
Signs of head trauma	37 (2.5%)	15 (3.0%)	0.83 (0.45-1.52)	0.541
Laceration	14 (2.1%)	38 (2.8%)	0.75 (0.40-1.39)	0.355
Hematoma	19 (2.6%)	33 (2.6%)	1.01 (0.57-1.78)	0.982
Abrasion	8 (2.1%)	44 (2.7%)	0.77 (0.36-1.65)	0.504
Abnormal neurologic findings	4 (3.4%)	45 (2.4%)	1.41 (0.50-3.98)	0.518
Blood alcohol level >100 mg/dl	3 (12.5%)	0 (0%)	-	0.130
Trauma activation	8 (2.1%)	48 (2.7%)	0.74 (0.35-1.59)	0.442
Arrived by ambulance	40 (2.7%)	16 (2.4%)	1.11 (0.62-1.99)	0.737
Loss of consciousness	2 (1.0%)	33 (2.6%)	0.38 (0.09-1.61)	0.175
Headache	10 (1.9%)	24 (2.6%)	0.73 (0.35-1.54)	0.403
Nausea	0 (0%)	33 (2.5%)	-	0.180
Vomiting	1 (3.6%)	35 (2.5%)	1.47 (0.19-11.14)	0.706
Dizziness	4 (2.9%)	25 (2.5%)	1.19 (0.41-3.48)	0.747

## Discussion

Geriatric patients with a fall-related head injury who presented to the ED without requiring hospital admission exhibited a short-term ED return rate of 14.1%. Of these patients who had a repeat ED visit, most returned due to sequelae of the initial fall (58.6%); however, a smaller subset of patients (18.5%) returned with a new traumatic injury, mostly caused by a second fall. The rate of ED returns is concerning and may be reduced by implementing post-discharge interventions [10]. Among all geriatric fall patients, return ED visits may be decreased by an increased emphasis on the need for outpatient primary care follow-up and post-discharge planning to address persisting complaints resulting from the initial injury. Outpatient management of these patients should also include implementing a coordinated care plan of fall prevention strategies, such as the STEADI program, to reduce recurrent falls [10].

While broad implementation of these strategies to all patients discharged from the ED would be ideal, those at higher risk of return visits should be targeted. No basic demographic factors including age, gender, or race predisposed patients to a greater rate of ED returns due to a fall within 14 days after the initial ED presentation. However, our study found that a past medical history of either dementia or stroke was associated with an increased rate of repeat ED visits within 14 days. We hypothesize that the neurologic aspects of balance, decision-making, and spatial perception play a significant role in recidivism within this brief period post-ED presentation. Interestingly, several recognized risk factors for falls were not found to be significant in our study. These include diabetes, hypertension, coronary artery disease, congestive heart failure, cancer, COPD, atrial fibrillation, deep vein thrombosis, peripheral vascular disease, and heart valve replacement [4]. Unexpectedly, tobacco use was also a risk factor for a 14-day ED return. The reason for this is unclear and may relate to increased neurovascular fragility due to tobacco use [11]. Likewise, as tobacco use increases the risk of stroke and dementia [12,13], there may be a secondary mechanism for tobacco use increasing repeat fall risk.

As the identified factors associated with an increased risk of ED return within 14 days relate to mentation and cognition rather than physical strength or mechanical stability, proposed fall-prevention strategies should focus on the neurological components contributing to fall risk. In older adults, there is a 10% risk of injury per fall, which accounts for 10.3% of all diagnoses requiring later readmission [8,14,15]. Given the significance of falls in the geriatric population and the limited success of strategies for decreasing repeat fall risk, we propose consideration of tobacco use and a history of dementia or stroke in determining future fall risk for older patients presenting to the ED with a fall-related injury.

Limitations

One limitation of our study is the reliance on patient medical records as the source of information, which may be subject to omissions and inaccuracies. However, mistakes in documentation would not skew results in a specific direction. Additionally, potentially confounding variables may not have been accounted for during data collection. Factors such as frailty and prior fall history are likely associated with an increased rate of new falls and return ED visits, but these items were not assessed as part of this study. Another limitation is that patients may have had subsequent ED visits at other hospitals not involved in the study. While transferred patients were excluded from enrollment during the index visit, patients with any significant injury due to a repeat fall would likely have been transferred to one of these two trauma centers and captured in our data.

## Conclusions

The sizable number of patients returning to the ED following fall-related head trauma is concerning, particularly for patients who are identified with dementia, stroke, and tobacco use. Given the attainability of these risk factors, the findings of this study provide an effective method of identifying at-risk patients to apply targeted prevention strategies. In the immediate period after a fall, enhanced outpatient follow-up or risk mitigation strategies should be considered to decrease further morbidity. Further research may investigate the success of utilizing these interventions to reduce return ED visits.
